# Prescription patterns of psychotropics for adults treated with ADHD medications: Analysis of the National Database of Health Insurance Claims and Specific Health Checkups of Japan (NDB) of 2019

**DOI:** 10.1002/pcn5.147

**Published:** 2023-09-26

**Authors:** Kanako Ishizuka

**Affiliations:** ^1^ Health Support Center Nagoya Institute of Technology Aichi Japan

The use of psychotropic polypharmacy, defined as the use of medication from two or more psychotropic classes, is quite common in clinical practice despite limited evidence of efficacy and mounting safety concerns. Attention‐deficit/hyperactivity disorder (ADHD) is one of the most disabling and common psychiatric disorders, persisting from childhood to adulthood. Individuals with ADHD are known to have many comorbid psychopathologies, making them candidates for psychotropic polypharmacy.^1^ In Japan, the approved psychotropic medications and the diagnosed disorders differ between pediatric and adult populations. Therefore, this study aimed to explore the utilization of multiple classes of psychotropics among patients undergoing ADHD pharmacotherapy, specifically targeting the adult population in Japan.

A retrospective study was conducted using the National Database of Health Insurance Claims and Specific Health Checkups of Japan (NDB), which covered 99.9% of public health insurance claims from hospitals, 97.9% from clinics, and 99.9% from pharmacies as of May 2015. The sampling data set of outpatients consisted of 1% of all claims data that were randomly extracted based on standard demographic characteristics in terms of age and sex in Japan, and included anonymized data on diagnoses, prescribed medications, examinations conducted, age group, and sex. Since the sensitivity of diagnoses in the database is generally low,^2^, ^3^ this study focused on pharmacotherapy. Data on individuals aged 20 years and over prescribed any of three ADHD medications as of October 2019 were retrieved from the NDB. The number of psychotropic medications prescribed concurrently with ADHD medications—atomoxetine, guanfacine, and the osmotic‐controlled‐release oral delivery system methylphenidate—was identified. The drug classification, including antipsychotics, antidepressants, anxiolytics/hypnotics, and mood stabilizers, is shown in Supporting Information: Table S1. DB Browser for SQLite Version 3.12.2 for macOS (https://sqlitebrowser.org) was used for analysis.

Less than half of individuals in their 20s and nearly 20% of those over age 30 were prescribed ADHD medications alone. Among patients in their 20s, anxiolytics/hypnotics, antidepressants, and antipsychotics were prescribed at similar rates, while among those aged 30 and above, antidepressants and anxiolytics/hypnotics were more frequently prescribed (Table 1). Based on these observations, two potential scenarios emerge. The first one is that ADHD medications were prescribed to those who meet the diagnostic criteria for ADHD. In this case, adults with ADHD might exhibit multiple psychiatric symptoms to require polypharmacy of psychiatric medications. The second scenario is that pharmacotherapy for ADHD was being administered to individuals who might not fully meet the ADHD diagnostic criteria, but who are experiencing specific symptoms, such as issues with attention, impulsivity, and hyperactivity. The author and colleagues revealed that the levels of adult patients' self‐perceived ADHD characteristics were significantly associated with the severity of depressive symptoms,^4^ indicating that diagnosing ADHD by the self‐administered questionnaire alone might overestimate its prevalence, especially when the depressive state becomes severe. Before considering prescribing ADHD medications, an assessment of the patient's core psychiatric problem is essential to avoid unnecessary polypharmacy. In any case, the use of psychotropic combinations might be justified as long as prescribed by well‐trained providers.^5^ According to a survey using the NDB, approximately 40% of individuals with ADHD are newly diagnosed after age 19 years.^6^ Considering that over 70% of Japanese patients prescribed psychotropic medications receive either a single or two‐drug prescription,^7^ and that the prescription rates of anxiolytics/hypnotics remained high despite four medical fee reductions in reimbursement between 2012 and 2018 to promote the appropriate use of psychotropic drugs in Japan,^8^ further research is needed to investigate the risk factors and explore preventive interventions for adults who require polypharmacy involving ADHD medications and other psychotropic drugs.

**Table 1:** Number of psychotropic medications concurrently with ≥1 ADHD medication prescription and frequency of specific medication classes prescribed to those receiving ≥1 psychotropic medications in addition to ADHD medications.

Predicted *N*		20−29 years	≥30 years
		*N* = 29,841	*N* = 61,246
Number of psychotropic medications concurrently with ≥1 ADHD medication prescription, %			
0		45	23
1		26	18
2		15	18
3		10	16
≥4		4	26
Frequency of specific medication classes prescribed to those receiving ≥ psychotropic medications in addition to ADHD medications, %			
Antidepressants		49	57
Anxiolytics/hypnotics		56	71
Antipsychotics		46	44
Mood stabilizers		20	29

Abbreviation: ADHD, attention‐deficit/hyperactivity disorder.

There are several limitations to the present study. First, this cross‐sectional study might have overestimated polypharmacy since it did not account for unintended polypharmacy resulting from medication switching. Second, information regarding the medication adherence was not available in this data set. If a patient did not take the prescribed medication as directed by the physician, additional medications might be added due to insufficient symptom improvement. Third, the entire population was not accounted for by the NDB, which comprised 1% of all claims data. Nevertheless, this study provides representative evidence of the prescription patterns of psychotropics with ADHD medications in Japan. Whether psychotropic polypharmacy in adulthood with ADHD symptoms is excessive, appropriate, or insufficient is still under debate and requires further longitudinal evaluation. Furthermore, evidence of the efficacy and safety is needed to guide psychotropic polypharmacy practice.

## AUTHOR CONTRIBUTIONS

Kanako Ishizuka conceived of conception and design of the study, performed the data collection and data analysis, and drafted the manuscript and figure.

## CONFLICT OF INTEREST STATEMENT

The author declares no conflicts of interest.

## ETHICS APPROVAL STATEMENT

N/A

## PATIENT CONSENT STATEMENT

N/A.

## CLINICAL TRIAL REGISTRATION

N/A.

## Supporting information

Supporting information.

## Data Availability

N/A
